# Supplementary material for: OncoRisk: a state-of-the-art web server for bridging the oncogenic databases and pan-cancer cohorts to the translational oncology
Source: Commun Biol. 2026 Apr 8;9:519. doi: 10.1038/s42003-026-10005-5 (PMC13068978; doi:10.1038/s42003-026-10005-5)
Supplement: Supplementary file 2 — Supplementary Information [file 42003_2026_10005_MOESM2_ESM.pdf]

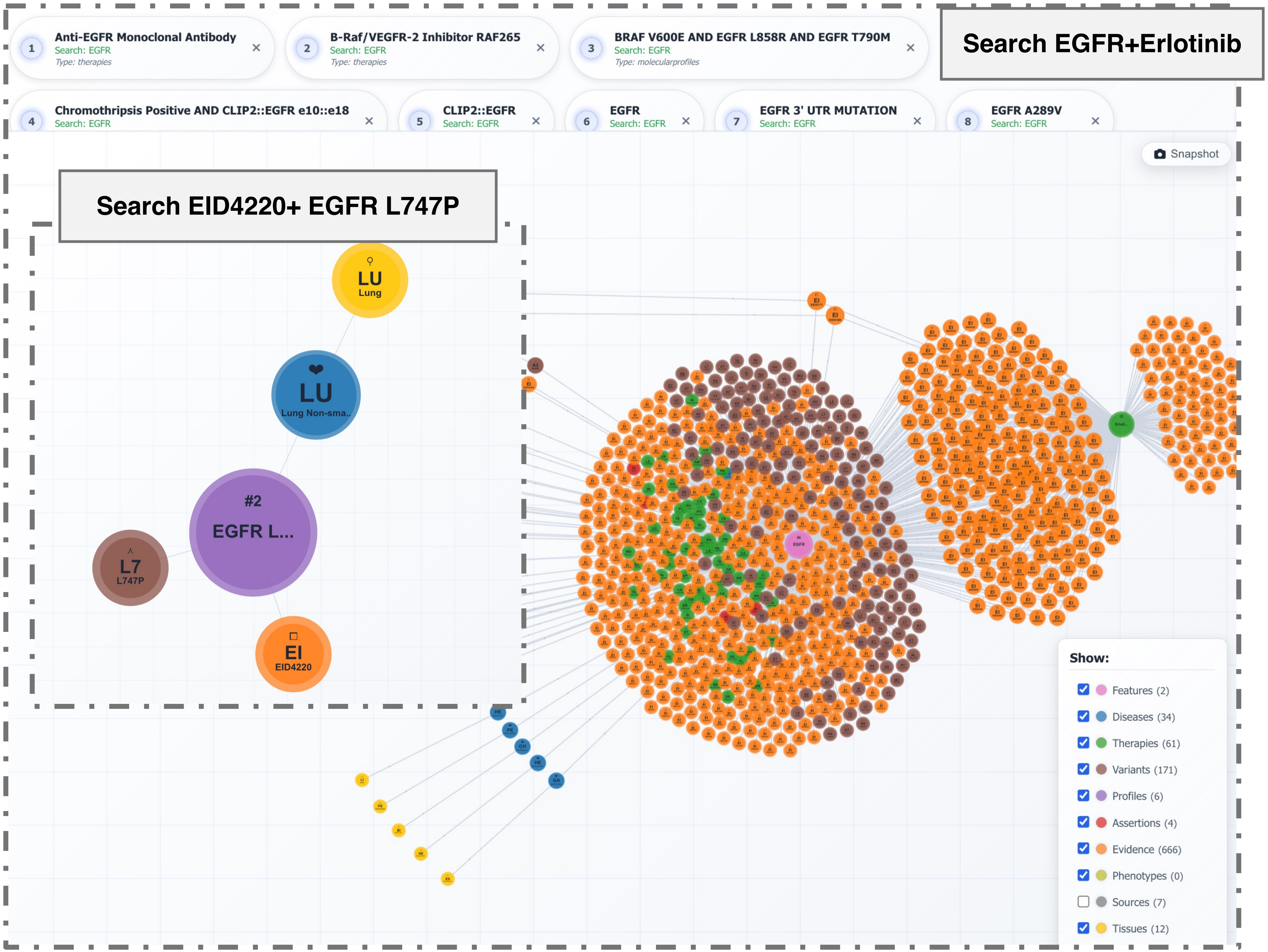

Anti-EGFR Monoclonal Antibody
Search: EGFR
Type: therapies
B-Raf/VEGFR-2 Inhibitor RAF265
Search: EGFR
Type: therapies
BRAF V600E AND EGFR L858R AND EGFR T790M
Search: EGFR
Type: molecularprofiles
Search EGFR+Erlotinib
Chromothripsis Positive AND CLIP2::EGFR e10::e18
Search: EGFR
CLIP2::EGFR
Search: EGFR
EGFR
Search: EGFR
EGFR 3' UTR MUTATION
Search: EGFR
EGFR A289V
Search: EGFR
Snapshot
Search EID4220+ EGFR L747P
LU
Lung
LU
Lung Non-sma..
#2
EGFR L...
L7
L747P
EI
EID4220
Show:
Features (2)
Diseases (34)
Therapies (61)
Variants (171)
Profiles (6)
Assertions (4)
Evidence (666)
Phenotypes (0)
Sources (7)
Tissues (12)

Supplementary Figure 1. Search results from Network tools for EGFR+Erlotinib, and further investigation on EID4220 and EGFR L747P. The interactive network visualization demonstrates a robust connection between Erlotinib and EGFR, supported by 666 evidence records (orange nodes) retrieved from the CiVIC database. Detailed inspection of specific nodes, such as Evidence Item EID4220, reveals that the EGFR L747P mutation is associated with a poor clinical response (resistance) to Erlotinib on the evidence's details page.

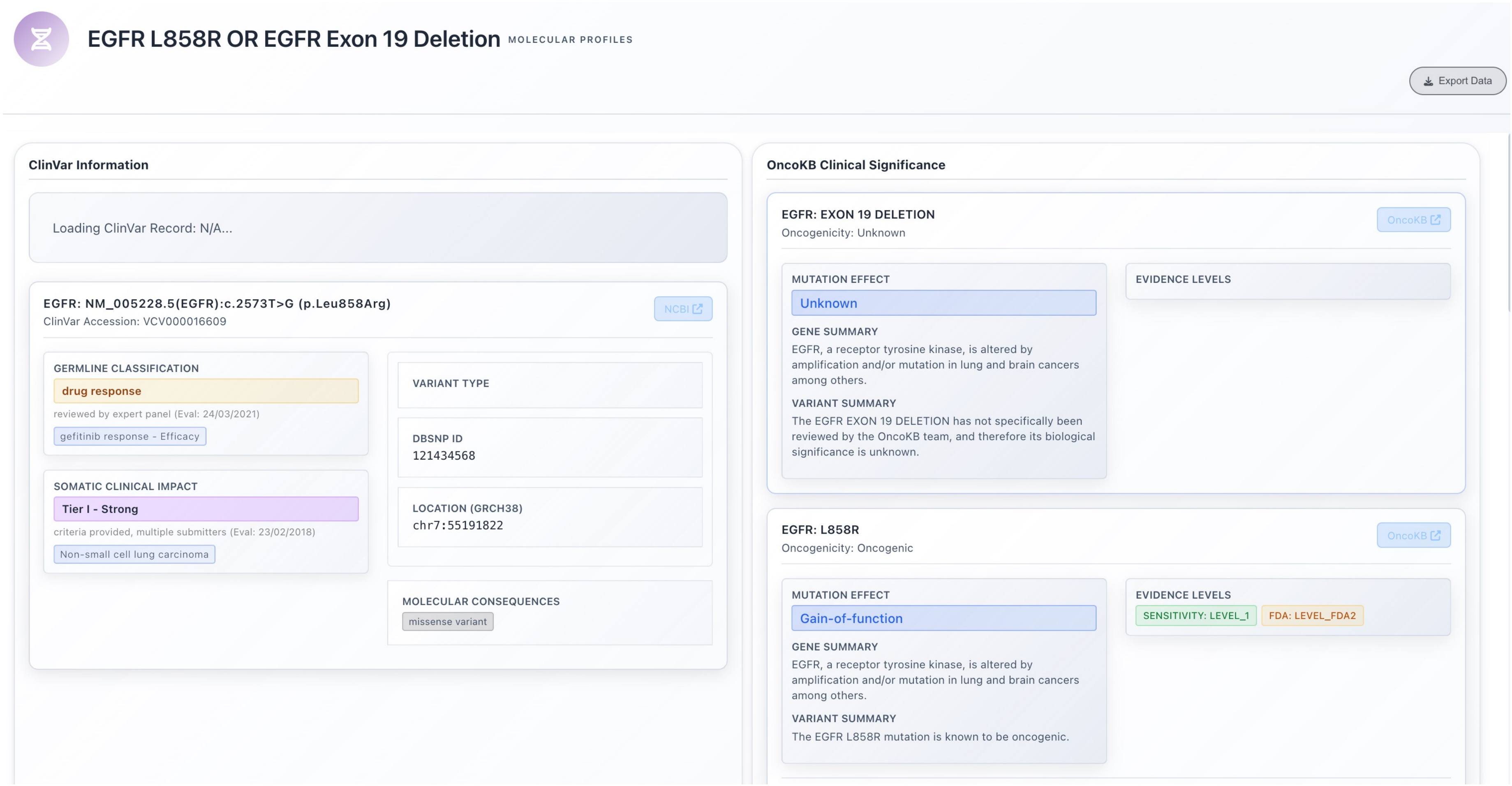

Supplementary Figure 2. Gain-of-function and Level 1 evidence from OncoKB, together with Tier 1-strong attribute from ClinVar, is displayed from the associated molecular profiles “EGFR L858R OR EGFR Exon 19 Deletion.

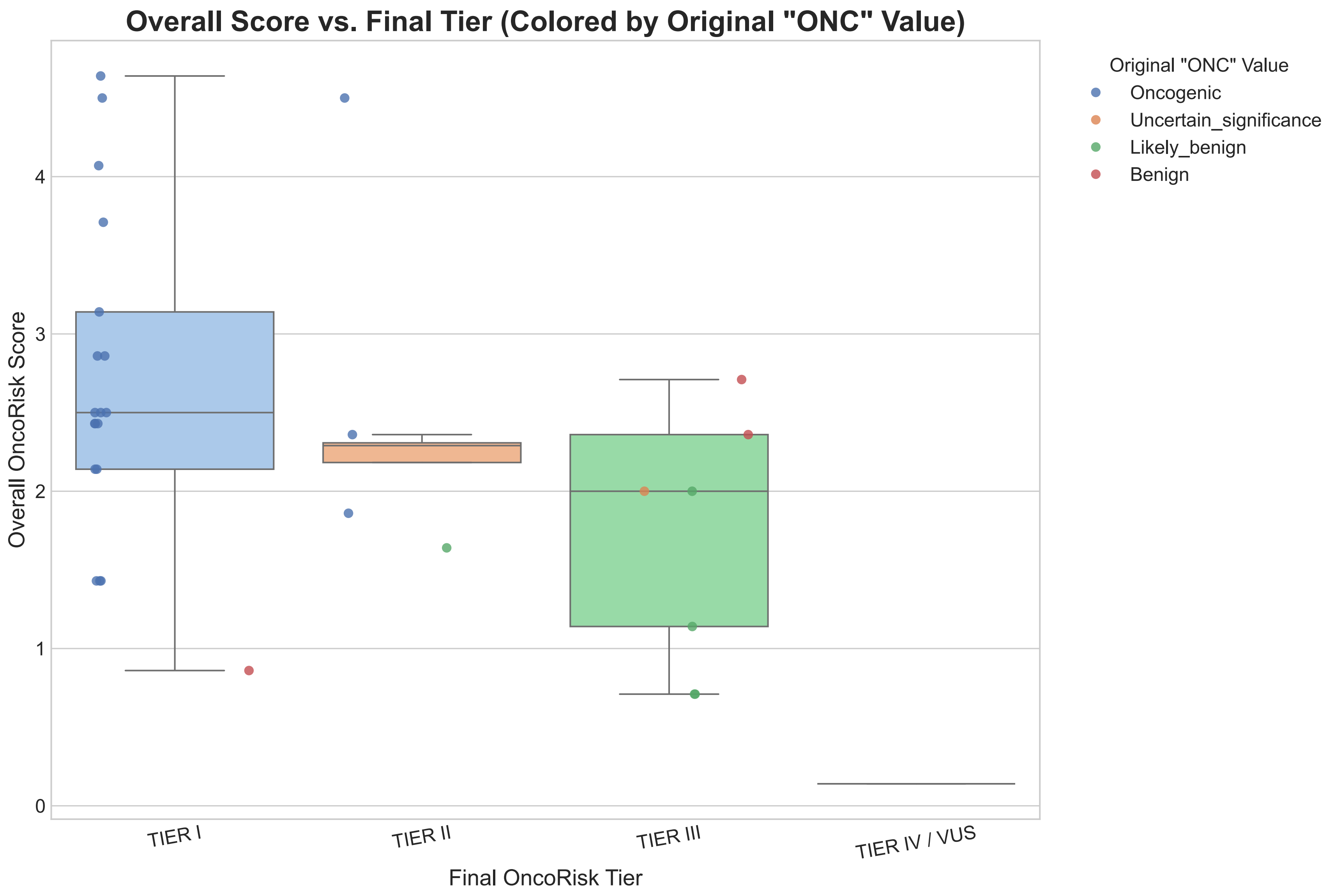

Supplementary Figure 3. Validation of OncoRisk Final Tier and scores compared to the original ONC values from ClinVar. The majority of oncogenic mutations from the positive control group are correctly prioritized as Tier 1, with their final quantitative scores reflecting the depth of integrated evidence. The assignment of certain variants to Tier 2 demonstrates the system's refined evidence-weighting adjustments. Conversely, most benign and likely benign variants from the negative control group are classified as Tier 3.

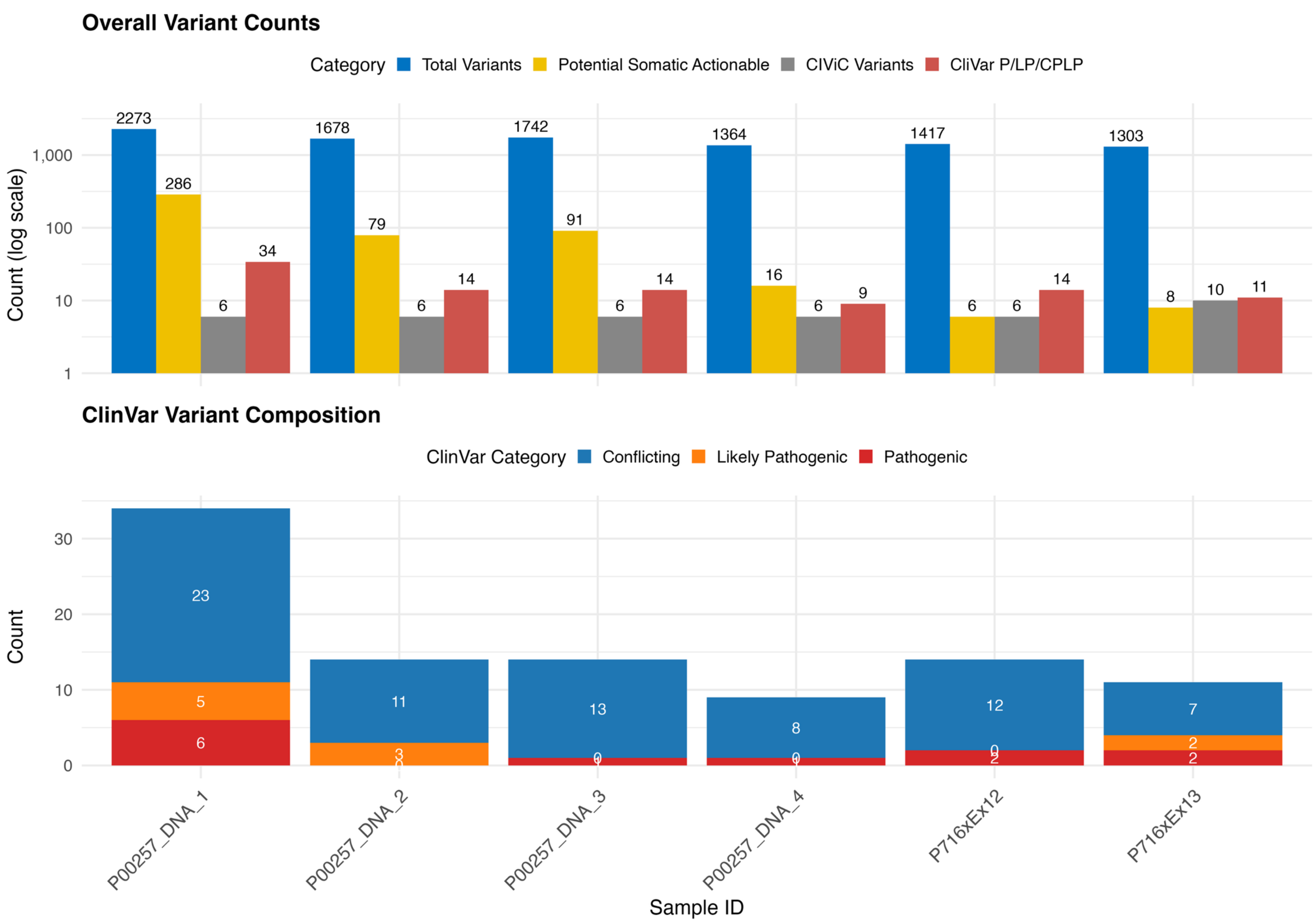

Overall Variant Counts
Category
Total Variants
Potential Somatic Actionable
CIViC Variants
CliVar P/LP/CPLP
Count (log scale)
1,000
100
10
1
2273
286
6
34
1678
79
6
14
1742
91
6
14
1364
16
6
9
1417
6
6
14
1303
8
10
11
ClinVar Variant Composition
ClinVar Category
Conflicting
Likely Pathogenic
Pathogenic
Count
30
20
10
0
23
5
6
11
3
0
13
0
1
8
0
1
12
0
2
7
2
2
P00257_DNA_1
P00257_DNA_2
P00257_DNA_3
P00257_DNA_4
P716xEx12
P716xEx13
Sample ID

Supplementary Figure 4. Pipeline-analysis results for 6 unique Demo somatic profiling samples. The top panel compares the total number of variants for each sample against key clinical and actionable subsets, including total variants, potential somatic actionable variants (somatic mutations with high-impact, oncogenic, or ClinVar pathogenic/likely pathogenic status), variants with CIViC evidence, and the sum of all ClinVar pathogenic, likely pathogenic, and conflicting variants. The bottom panel shows the composition of ClinVar variants for each sample.

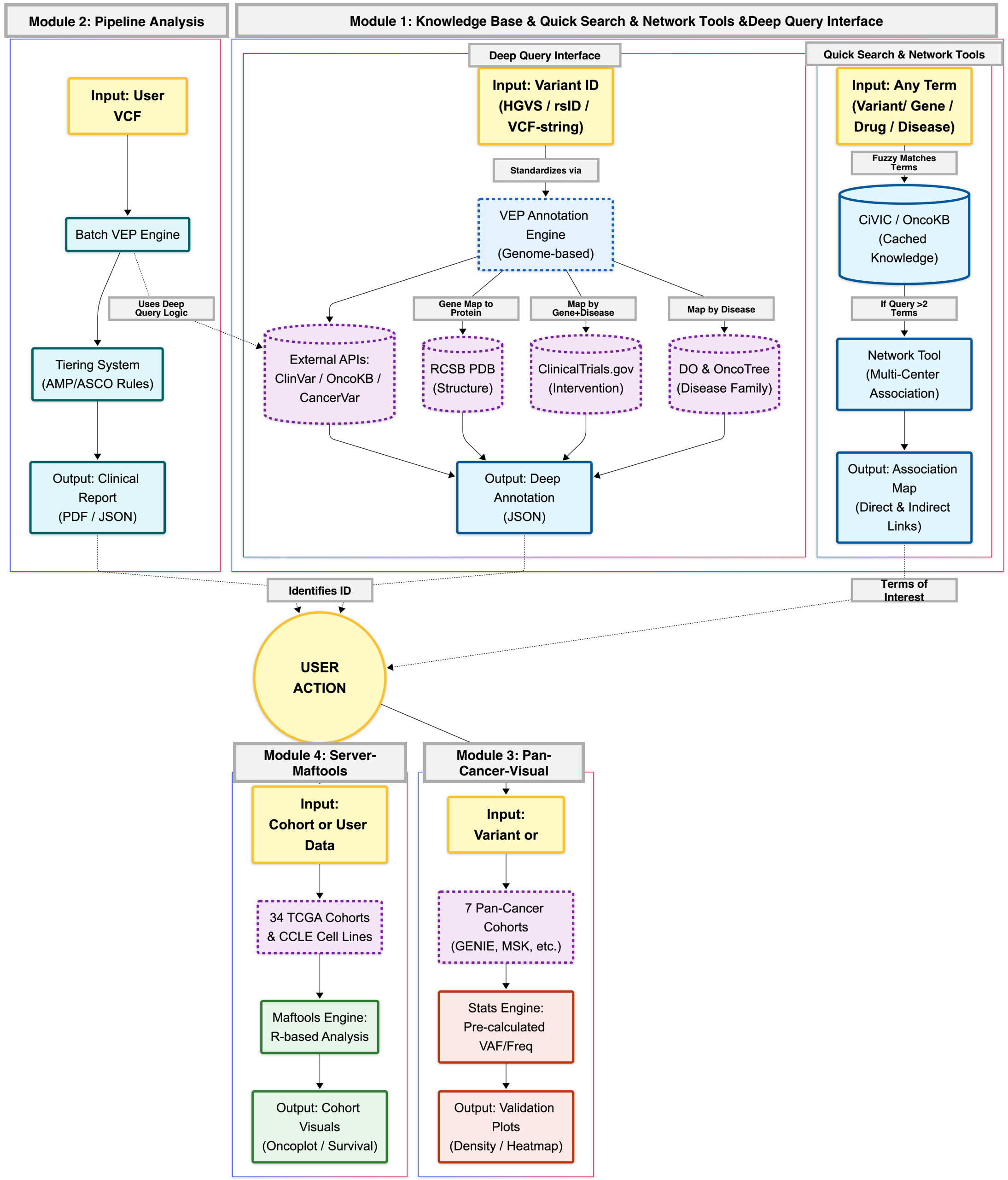

Module 2: Pipeline Analysis
Module 1: Knowledge Base & Quick Search & Network Tools &Deep Query Interface
Input: User VCF
Batch VEP Engine
Uses Deep Query Logic
Tiering System (AMP/ASCO Rules)
Output: Clinical Report (PDF / JSON)
Deep Query Interface
Input: Variant ID (HGVS / rsID / VCF-string)
Standardizes via
VEP Annotation Engine (Genome-based)
Gene Map to Protein
Map by Gene+Disease
Map by Disease
External APIs: ClinVar / OncoKB / CancerVar
RCSB PDB (Structure)
ClinicalTrials.gov (Intervention)
DO & OncoTree (Disease Family)
Output: Deep Annotation (JSON)
Quick Search & Network Tools
Input: Any Term (Variant/ Gene / Drug / Disease)
Fuzzy Matches Terms
CiVIC / OncoKB (Cached Knowledge)
If Query >2 Terms
Network Tool (Multi-Center Association)
Output: Association Map (Direct & Indirect Links)
Identifies ID
Terms of Interest
USER ACTION
Module 4: Server-Maftools
Module 3: Pan-Cancer-Visual
Input: Cohort or User Data
34 TCGA Cohorts & CCLE Cell Lines
Maftools Engine: R-based Analysis
Output: Cohort Visuals (Oncoplot / Survival)
Input: Variant or
7 Pan-Cancer Cohorts (GENIE, MSK, etc.)
Stats Engine: Pre-calculated VAF/Freq
Output: Validation Plots (Density / Heatmap)

Supplementary Figure 5. Entity diagram for illustrating OncoRisk's database integration within all four main modules. The Server-Maftools and Pan-Cancer Visual is currently only linked to the main knowledge Base and interface of OncoRisk by the user's action of searching on the specific interest.

Sample_name	Cancer_type
P00257_DNA_1.hard-filtered.vcf.csv	Primary endometrioid carcinoma of endometrium of body
P00257_DNA_2.hard-filtered.vcf.csv	Endometriosis of uterus
P00257_DNA_3.hard-filtered.vcf.csv	Familial cancer of breast
P00257_DNA_4.hard-filtered.vcf.csv	Malignant tumor of breast
P716xEx12.hard-filtered.vcf.csv	stomach cancer
P716xEx13.hard-filtered.vcf.csv	pancreas cancer

Supplementary Table 1. Demo Sample metadata. This table summarizes the clinical diagnoses of the six tissue-derived DNA samples used as the Demo sample in the OncoRisk pipeline analysis. Each sample represents a distinct oncological diagnosis (with one endometriosis control) and underwent somatic tumor profiling using the Illumina TruSight (TS) 500 workflow. The resulting small variant sequencing data (VCF files) were subsequently processed through the automated OncoRisk interpretation pipeline.

Module	Data Source	Data Volume	Data Types & Key Attributes	Integration Role
**Knowledge Base & Quick Search; Deep Query &Pipeline Analysis**	**CiVIC**	Pre-cached Oncogenic terms of 9 main categories from API access; Downloaded VCF (20250630)	Assertions, evidence, molecular profiles, features, variants, disease, therapies, phenotypes, sources	Defines the backbone for oncogenic terms and variant mapping for main knowledge base interface
	**OncoKB**	Real-time API / caching	Oncogenicity, Mutation Effect, Therapeutic Evidences, FDA Level	Provides precision oncology therapeutic tiering and drug actionability
	**ClinVar**	Read-time API / caching & Downloaded VCF (2025 ClinVar (20250623)	Variant Germline Pathogenicity and Somatic Clinical impact (Tiers & Review status)	Validates clinical significance and complementing germline/somatic classification
**Deep Query & Pipeline Analysis**	**CancerVar**	Real-time API / caching	Oncogenicity scoring, Automated AMP/ASCO interpretation results	Provides AI-empowered automated scoring for mutations
	**ClinicalTrials.gov**	Real-time API / caching	Clinical trial status (Recruiting, Active), Phases, Study details	Links actionable variants to currently available clinical trials.
**Deep Query**	**Disease Ontology / OncoTree**	Full databases via downloaded files	Standardized disease nomenclature & disease hierarchy	Enables hierarchical disease queries and maps specific subtypes to broader cancer families and tissue-level grouping.
	**RCSB PDB**	Full databases via downloaded files	3D Protein-Ligand-mutant complex structures	Visualizes mutation locations relative to drug binding pockets.
**Pan-Cancer Visual**	**AACR Project GENIE (v18.0)**	250,018 samples	Somatic mutations profiles via different sequencing technologies, ethinities, cancer types and coverage	For querying sepecific variant in the cohort-somatic mutation profile & Pre-calculated Variant Allele Frequency (VAF) and real-world mutation prevalence and gene-based mutation prevalence
	**MSK-CHORD**	25,040 samples		
	**China Pan-Cancer**	10,194 samples		
	**PCAWG**	2,922 samples		
	**Other Cohorts (MSC, MSS, SUMMIT)**	890 samples (combined)		
**Server-Maftools**	**TCGA & CCLE**	33 TCGA Cohorts; 2,427 Cell Lines (DepMap 2024 Q2)	MAF files with Clinical metadata	Used in "Server-Maftools" for all analyiss and plotting

Supplementary Table 2. Overview of integrated database & cohort volume, type, and key attributes. In different modules, OncoRisk has integrated different oncogenic knowledge bases or pan-cancer cohorts.

Category	Feature	**OncoRisk**	cBioPortal	CancerVar	PORI	Onkopus	Tri©DB	PANDA	Oviz-Bio	OncoKB	CIViC	COSMIC	CGI
**System Accessibility**	**Platform Type**	**Web**	Web	Web	Web + local	Web + Docker	Web	Web	Web	Web	Web	Web	Web
	**Free Academic Access**	**✓**	✓	✓	✓ (open-source)	✓	✓	✓	✓	✓ (partial)	✓	✓ (research)	✓
	**Code-free for End Users**	**✓**	✓	✓	○	✓	✓	✓	✓	✓	✓	✓	✓
**Data Scope**	**Multi-database Integration**	**✓ (>10 Sources)**	○	✓	✓	✓	✓ (>30 DBs)	✓ (pathway/phenotype DBs)	○	○	○	○	✓
	**Pan-cancer Cohorts**	**✓ (7 cohorts, ~300k samples)**	✓ (>200 studies)	○	✓ (TCGA used for benchmarking)	○	○	○	○	○	○	✓	○
	**CNV / SV / Fusion Support**	**✓ (Basic)**	✓ (CNV)	✓ (CNV)	✓	✓	✓ (SNV/SV/MSI)	○	✓ (CNV/SV/fusion/virus)	○	○	✓	○
**Input & Processing**	**Raw VCF Input**	**✓**	○	✓	✓	✓	✓	○	✓	○	○	○	✓
	**Batch Analysis**	**✓ (High-throughput)**	✓	✓	✓	✓	✓	○	✓	○	○	○	✓
**Clinical Reporting**	**Automated Clinical Report (PDF / HTML)**	**✓ (PDF & JSON)**	○	✓ (Text summary))	✓	○	✓	○	○	○	○	○	○
	**Tiered Classification**	**✓ (Tier 1-4)**	○	✓	✓	✓	✓	○	○	✓	✓	○	✓
	**Therapy / Drug**	**✓**	○	✓	✓	✓	✓	○	○	✓	✓	✓ (resistance)	✓

	**Annotation**												
	**Clinical Trial Links**	**✓ (Real-time)**	○	○	✓	✓	✓	○	○	○	○	○	○
**Cohort Analytics**	**Survival Analysis**	**✓**	✓	○	○	○	○	○	○	○	○	○	○
	**Mutational Signatures**	**✓**	○	○	○	○	✓	○	✓	○	○	✓	○
	**Cohort comparisons**	**✓**	✓	○	○	○	○	○	○	○	○	○	○
**Visualization**	**Oncoplot /mutation Landscape**	**✓**	✓	○	✓	○	○	○	✓	○	○	○	○
	**Pathway / Network Views**	**✓ (Term Network)**	✓ (Network)	✓ (Pathways)	○	✓ (Network)	✓ (network + sunburst)	✓ (pathway-centric)	✓ (Multi-layer views)	○	○	○	○
	**3D Protein–Drug–Variant**	**✓**	○	○	○	✓ (AlphaFold-based)	○	○	○	○	○	○	○
**Summary**	**Main Focus**	**All-in-one** (knowledge + cohorts + analytics + reporting)	Cohort Explorer	Somatic Variant Interpretation	Reporting Platform	VUS & protein structure	Precision medicine knowledge base + Reporting	Pathway-level Visuals	Genomics Visuals	Curated Clinical KB	Community-curated clinical KB	Somatic Mutation Catalog	Automated oncogenomic interpretation

Supplementary Table 3. Comprehensive feature comparison of OncoRisk with other oncology bioinformatics platforms.
(✓: Supported / Available. ○: Not supported / Limited.) The table summarizes system accessibility, data scope, input and processing capabilities, clinical reporting functions, cohort-level analytics, and visualization features. Checkmarks indicate full support, open circles indicate partial or limited support, and empty cells indicate the absence of the feature based on publicly available documentation at the time of comparison. The focus of each platform reflects its primary design goal rather than exhaustive functionality.
