# Supplementary material for: OncoRisk: a state-of-the-art web server for bridging the oncogenic databases and pan-cancer cohorts to the translational oncology
Source: Commun Biol. 2026 Apr 8;9:519. doi: 10.1038/s42003-026-10005-5 (PMC13068978; doi:10.1038/s42003-026-10005-5)
Supplement: Supplementary file 3 — Description of Additional Supplementary Materials [file 42003_2026_10005_MOESM3_ESM.pdf]

Description of Additional Supplementary Files

File name: Supplementary Data 1

Description: Detailed breakdown of the multi-dimensional rule-based scoring system used to assign OncoRisk Tiers (I–IV).

File name: Supplementary Data 2

Description: A benchmarking analysis comparing OncoRisk's automated tier assignments to established clinical classifications from ClinVar (v20250623). A curated reference set (n=41) was stratified into: (1) a Positive Control Group, comprising ClinVar annotated "Oncogenic" variants (n=20) and SCI "Tier I - Strong" variants (n=5); and (2) a Negative Control Group, comprising ClinVar "Benign" or "Likely Benign" variants under ONC or SCI (Tier IV) standards (n=16). For each variant, the table provides the genomic identifier (CLNHGVS), the original ClinVar ONC/SCI status, the integrated OncoRisk final score, and the resulting Tier assignment. The data demonstrate that most oncogenic mutations were correctly prioritized as Tier 1, while scoring adjustments facilitated the stratification of Tier 2 variants. Benign variants were predominantly assigned to Tier 3.

File name: Supplementary Data 3

Description: Source data for Figure 5A,5B,5C,6D. Tab 1 (Figure 5A) includes the cohort metrics for sample size, total mutation counts, and distinct cancer type counts, alongside descriptive statistics (Mean, Median, Q1, and Q3) for mutation burden distributions. Tab 2 (Figure 5B) details the proportional representation of the top 10 most prevalent cancer 36 types and aggregated "Other" categories for each integrated cohort. Tab 3 (Figure 5C1) provides the numerical coordinates (x, y) for the Kernel Density Estimation of Variant Allele Frequency (VAF) distribution across four patient age groups in the GENIE cohort. Tab 4 (Figure 5C2) presents the Mean VAF matrix for the top 20 mutated genes across the top 30 cancer types in the GENIE cohort, including marginal mutation counts for cancer types and gene frequencies. Tab 5 (Figure 6D) contains the gene mutation burden data for the TCGA KIRC cohort validation, focusing on recurrent mutations occurring in >= samples categorized by variant classification.
